# Healthcare provider perspectives on integrating a comprehensive spine care model in an academic health system: a cross-sectional survey

**DOI:** 10.1186/s12913-024-10578-z

**Published:** 2024-01-23

**Authors:** Wren Burton, Stacie A. Salsbury, Christine M. Goertz

**Affiliations:** 1 Division of Preventive Medicine, Brigham and Women’s Hospital, Harvard Medical School, Boston, MA USA; 2 Osher Center for Integrative Health, Brigham and Women’s Hospital and Harvard Medical School, Boston, MA USA; 3 Palmer Center for Chiropractic Research, Palmer College of Chiropractic, Davenport, IA USA; 4 Implementation of Spine Health Innovations, Department of Orthopaedic Surgery, School of Medicine, 300 W. Morgan Street, Durham, NC 27701 USA; 5 Duke Clinical Research Institute, Musculoskeletal Research, Duke University, 300 W. Morgan Street, Durham, NC 27701 USA; 6 Duke-Margolis Center for Health Policy, Duke University, 300 W. Morgan Street, Durham, NC 27701 USA

**Keywords:** Low back pain, Clinical practice guidelines, Survey, Health systems, Chiropractic, Physical therapy, Spinal pain

## Abstract

**Background:**

Healthcare systems (HCS) are challenged in adopting and sustaining comprehensive approaches to spine care that require coordination and collaboration among multiple service units. The integration of clinicians who provide first line, evidence-based, non-pharmacological therapies further complicates adoption of these care pathways. This cross-sectional study explored clinician perceptions about the integration of guideline-concordant care and optimal spine care workforce requirements within an academic HCS.

**Methods:**

Spine care clinicians from Duke University Health System (DUHS) completed a 26-item online survey via Qualtrics on barriers and facilitators to delivering guideline concordant care for low back pain patients. Data analysis included descriptive statistics and qualitative content analysis.

**Results:**

A total of 27 clinicians (57% response) responded to one or more items on the questionnaire, with 23 completing the majority of questions. Respondents reported that guidelines were implementable within DUHS, but no spine care guideline was used consistently across provider types. Guideline access and integration with electronic records were barriers to use. Respondents (81%) agreed most patients would benefit from non-pharmacological therapies such as physical therapy or chiropractic before receiving specialty referrals. Providers perceived spine patients expected diagnostic imaging (81%) and medication (70%) over non-pharmacological therapies. Providers agreed that receiving imaging (63%) and opioids (59%) benchmarks could be helpful but might not change their ordering practice, even if nudged by best practice advisories. Participants felt that an optimal spine care workforce would require more chiropractors and primary care providers and fewer neurosurgeons and orthopedists. In qualitative responses, respondents emphasized the following barriers to guideline-concordant care implementation: patient expectations, provider confidence with referral pathways, timely access, and the appropriate role of spine surgery.

**Conclusions:**

Spine care clinicians had positive support for current tenets of guideline-concordant spine care for low back pain patients. However, significant barriers to implementation were identified, including mixed opinions about integration of non-pharmacological therapies, referral pathways, and best practices for imaging and opioid use.

**Supplementary Information:**

The online version contains supplementary material available at 10.1186/s12913-024-10578-z.

## Background

Comprehensive, sustainable, patient-centered approaches to spine care are an imperative to address the global burden of back and neck pain [1–3]. High-quality spine care relies on several crucial elements to achieve optimal outcomes: primary prevention; early intervention with guideline-concordant care; and access to specialty care when needed. Prevention plays a pivotal role in mitigating the burden of spinal pain by emphasizing public health measures, self-management and lifestyle modifications [4–6]. Early intervention, which may be most efficiently provided by primary spine practitioners (PSP) such as physical therapists and doctors of chiropractic, may avert or delay the progression of spinal disability through timely diagnoses and provision of evidence-based treatments [7–11]. Access to specialty care, including orthopedic surgeons, neurosurgeons, and physiatrists, among other health professionals, is necessary to support patients who require more intensive interventions. An integrated systems approach, encompassing a broad range of services, provided by a multidisciplinary workforce, and tailored to individual patient needs, offers a promising framework to optimize spine care delivery and patient outcomes [12–15].

Such comprehensive approaches for patients with spine pain acknowledge that effective treatment extends beyond the traditional boundaries of individual clinical encounters, and requires coordination, integration, and collaboration among several components of the healthcare system. Moreover, the use of up-to-date clinical practice guidelines (CPGs) makes evidence-based decision-making more likely [14]. However, meaningful uptake of CPGs for multidisciplinary spine care is limited [16, 17]. Comprehensive spine care pathways that incorporate evidence-based CPGs demonstrate cost-effectiveness in large healthcare systems [18, 19]. Yet, little is known about how physicians and other primary care providers view spine care pathways in these settings [19–22]. Furthermore, there is a lack of information about non-pharmacologic treatments (NPT) and spine care practices in United States within private healthcare settings, and a need for a better understanding of how they are integrated into usual medical practices [22–24]. While PSPs have the potential to positively impact clinical and costs outcomes, these practitioners are often constrained from full participation in integrated healthcare teams by such barriers as limited insurance reimbursement, exclusion from documentation in electronic health records (EHR), and referral patterns which position PSPs as practitioners of last resort, rather than as first line options [11, 19, 23, 25–27].

The goal of this effort was to better understand healthcare clinician perceptions of potential barriers and facilitators to the integration of guideline-concordant spine care services for patients with low back pain (LBP) at Duke University Health System (DUHS). The overall purpose was to create new knowledge while simultaneously laying the groundwork required to develop and implement the Duke Spine Health Program—an interdisciplinary, evidence-based, patient-centered model for spine care delivery, with a focus on LBP.

## Methods

We administered a cross-sectional online survey to practicing spine care providers within DUHS between July 15 and August 10, 2021. The study was conducted in accordance with the Declaration of Helsinki and approved by the DUHS Institutional Review Board on June 25, 2021 (Protocol ID: Pro00108441). All participants provided informed consent via the survey platform. We report study findings using the Strengthening the Reporting of Observational studies in Epidemiology (STROBE) guidelines checklist in Supplementary File 1 [28].

### Setting and sample

DUHS is an integrated academic health system located within the Raleigh-Durham area of North Carolina, USA. DUHS has approximately 25,000 employees and offers a full range of inpatient and outpatient clinical services, including both primary and specialty care. In the year that this study was conducted, patients paid DUHS hospitals and clinics more than 4.7 million visits, the vast majority for outpatient visits. The Duke Spine Division coordinates care between the Departments of Orthopaedic and Neurosurgery. Forty clinicians within the Division were invited to participate in the survey, as well as four physical therapists serving in the PSP role, one chiropractor, and two primary care physicians who were actively involved in program implementation (*n* = 47). No exclusions to participation were applied to these clinicians.

### Instrument

The 26-item survey focused on barriers and facilitators to delivering interdisciplinary, guideline-concordant care to patients with spine-related disorders, included 10 additional demographic questions and matrices of Likert scale items that focused on five domains considered essential for program success. Survey questions were developed by a multidisciplinary team to address our specific programmatic and research objectives (Supplementary File 2). The relevance of survey content was ensured through the expertise of the team, with review for face validity by Division personnel, and its ease of use was pre-tested by team members. However, the survey was not formally validated due to practical constraints within the project timeline. Stepped care items (*n* = 7) included facilitated self-care; individualized primary care; and specialty care, including consultation, advanced diagnostics, injections, and surgery. Resources (*n* = 9) include referral, information sources, and patient expectations about spine care. The benchmarking performance domain (*n* = 5) considered efforts to standardize delivery of spine care services across clinicians. The guideline concordant care domain (*n* = 3) evaluated clinicians’ perceived use of spine care CPGs in daily practice, with frequency rated as *every visit, often, infrequently,* or *never*. The final domain, optimal spine care workforce, included 2, simple card sort grids [29]. The first grid asked respondents to identify ideal members of a multidisciplinary spine care team: 1) first contact clinicians, 2) clinicians who should evaluate spine care patients when red flags are present or if NPTs do not achieve desired outcomes, and 3) clinicians who should play supportive roles for spine care patients. The second grid asked respondents to identify if the number of clinicians in the DUHS current workforce was optimal for providing spine care services, if more clinicians were needed, or if fewer clinicians was optimal. Respondents could also enter their thoughts about the topic of spine care into open-ended text boxes. Provider perceptions of healthcare costs also were gathered for administrative planning purposes but are not reported due to the proprietary nature of these data.

### Data collection

The survey was administered using Qualtrics (www.qualtrics.com), a secure web-based platform for data collection. Participants were contacted via email and provided a unique link to access the survey. Participants were informed that study participation was voluntary, and all responses were confidential. Responses were de-identified by a project team member before results were analyzed.

### Data analysis

Descriptive statistics summarized the data. Frequencies and percentages were reported for categorical variables and means and standard deviations (or median and interquartile ranges (IQR)) were reported for continuous variables. Answers from the short-answer, open-ended questions were reviewed as qualitative results and coded for common themes using conventional content analysis whereby themes are pulled directly from the text [30].

## Results

### Demographics

Table 1 presents respondent demographics. Forty-seven individuals, including all faculty of the Duke Spine Division and key program stakeholders, were invited; 27 persons responded in part; and 23 recorded complete responses to the survey, resulting in a response rate of 57%. Respondents were most likely to be male (*n* = 19, 70%), medical doctors (*n* = 16, 59%), worked in neurosurgery (*n* = 13, 48%) or orthopaedics (*n* = 9, 33%), and worked in their profession for more than 10 years (*n* = 15, 63%). Seven respondents reported completing a fellowship in spine surgery.

**Table 1:** Demographics of survey respondents^a^

Variable (# of responses)	Categories	*n (%)*
Age, Years (*n* = 27)	25–34	5 (19%)
	35–44	8 (31%)
	45–54	3 (12%)
	55–64	8 (31%)
	65–75	2 (8%)
	Median (IQR)	46 (27–57)
Gender (*n* = 27)	Male	19 (70%)
	Female	5 (19%)
	Prefer to not answer	3 (11%)
Clinical Degree^b^ (*n* = 27)	Medical Doctor (MD)	16 (59%)
	Physician Assistant (PA)	8 (30%)
	Physical Therapist (PT)	3 (11%)
	Doctor of Chiropractic (DC)	0
Clinical Department^b^	Neurosurgery	13 (48%)
	Orthopaedics	9 (33%)
	Physical Medicine & Rehabilitation	2 (7%)
	Pain Management	1 (4%)
	Physical or Occupational Therapy	2 (7%)
Clinical Setting^b^	Private Diagnostic Outpatient Clinic	17 (38.6%)
	Hospital Based Care	17 (38.6%)
	Inpatient Care	10 (22.7%)
Occupation^b^	Researcher	2 (6.3%)
	Clinician	23 (71.9%)
	Professor/Educator	7 (21.9%)
	Administrator	0
Years in Profession (*n* = 24)	0–3 years	2 (8%)
	4–10 years	7 (29%)
	10 + years	15 (63%)
Spine Surgery Fellowship(*n* = 24)	Completed	7 (29%)

^a^The number of responses to individual questions varied and percentages reflect the value from the total number of responses to the individual question

^b^Respondents could select all that apply

### Stepped care

Nearly all survey completers (*n* = 22, 81%) answered that patients would benefit from more access to NPTs such as physical therapy or chiropractic care (Table 2). These respondents also answered that primary care clinicians should recommend NPTs before referring to specialty care (*n* = 19, 70%). A majority replied that clinical pathways were not difficult to implement or sustain. However, fewer clinicians felt confident in their ability to refer patients to self-care programs.

**Table 2:** Survey results for domain 1 (stepped care), domain 2 (resources), and domain 3 (benchmarking performance)

*Question*	*Strongly Disagree*	*Disagree*	*Agree*	*Strongly Agree*
***Domain 1: Stepped Care (n = 24)***				
Patients in my clinical area would benefit from increased access to conservative approaches to spine care, such as physical therapy and chiropractic	1 (4.2%)	1 (4.2%)	7 (29.2%)	15 (62.5%)
Evidence-based spine care pathways are commonly followed in DUHS	3 (12.5%)	6 (25%)	9 (37.5%)	6 (25%)
Clinical care pathways are too difficult to implement and/or sustain in DUHS	4 (16.7%)	13 (54.2%)	3 (12.5%)	4 (16.7%)
DUHS has methods in place to support coordinated multidisciplinary care for spine patients	2 (8.3%)	7 (29.2%)	11 (45.8%)	4 (16.7%)
Primary care providers should recommend physical therapy before referring to specialty care	0 (0%)	2 (8.3%)	11 (45.8%)	11 (45.8%)
Primary care providers should recommend non-pharmacological spine care, such as yoga, massage, and chiropractic, before referring to specialty care	1 (4.2%)	4 (16.7%)	13 (54.2%)	6 (25%)
I know how to refer patients to self-care programs, such as yoga, exercise, and weight loss, within DUHS	2 (8.3%)	7 (29.2%)	10 (41.7)	5 (21%)
***Domain 2: Resources (n = 23)***				
DUHS provides access to the full range of services needed by spine care patients in our community	1 (4.3%)	6 (26.1%)	14 (60.9%)	2 (8.7%)
I wish I had more resources to support me in making referrals for spine care patients in my practice	1 (4.3%)	7 (30.4%)	10 (43.5%)	5 (21.7%)
I need more information about non-pharmacological care to integrate this into my practice	1 (4.3%)	15 (65.2%)	7 (30.4%)	0 (0%)
I need more information about community resources for patients with spine conditions	1 (4.3%)	6 (26.1%)	13 (56.5%)	3 (13%)
I feel like the administrative insurance processes (i.e. benefits and authorization) are a barrier to my patient's care	0 (0%)	5 (21.7%)	13 (56.5%)	5 (21.7%)
Most patients expect to receive diagnostic imaging as part of their spine care treatment	0 (0%)	1 (4.3%)	13 (56.5%)	9 (39.1%)
Most patients expect to receive medication as part of their spine care treatment	0 (0%)	4 (17.4%)	13 (56.5%)	6 (26.1%)
Most patients expect to receive physical therapy as part of their spine care treatment	0 (0%)	8 (34.8%)	14 (60.9%)	1 (4.3%)
Most patients expect to receive chiropractic care as part of their spine care treatment	2 (8.7%)	20 (87%)	1 (4.3%)	0 (0%)
***Domain 3: Benchmarking Performance (n = 23)***				
DUHS places too much emphasis on specialty care, such as surgery and injections, for spine patients	1 (4.3%)	12 (52.2%)	7 (30.4%)	3 (13%)
I am comfortable with my imaging ordering information being shared among providers in my division	0 (0%)	2 (8.7%)	12 (52.2%)	9 (39.1%)
I would reconsider some imaging requests if I knew my imaging order volume was substantially higher than my colleagues	1 (4.3%)	13 (56.5%)	7 (30.4%)	2 (8.7%)
I would reconsider some opioid prescribing if I knew my opioid prescribing volume was substantially higher than my colleagues	2 (8.7%)	5 (21.7%)	10 (43.5%)	6 (26.1%)
I am likely to give my imaging order a second thought if I see a Best Practice Advisory	3 (13%)	9 (39.1%)	10 (43.5%)	1 (4.3%)

### Resources

Most survey completers perceived that spine care patients expected to receive diagnostic imaging (*n* = 22, 81%) and pain medication (*n* = 19, 70%). Approximately the same number of respondents (*n* = 22, 81%) answered that patients do not expect to receive chiropractic care. About two-thirds answered that patients expect to receive physical therapy. There was largely a consensus from respondents that the healthcare setting provides access to the full range of services required by spine care patients, but they expressed a desire for more information about community resources, including for patient referrals. Additionally, most respondents (*n* = 18, 67%) answered that administrative processes were a barrier to care (Table 2).

### Benchmarking performance

Almost all survey completers (*n* = 21, 78%) were comfortable sharing their imaging ordering information among clinicians in their division (Table 2). While nearly half would likely reconsider an imaging order if they encountered a best practice advisory, a larger proportion responded that they would not necessarily reconsider imaging requests if they knew their order volume was substantially higher than that of their colleagues. Alternatively, more than two-thirds responded that they would reconsider some opioid prescribing if they knew their rates were substantially higher than their colleagues.

### Guideline concordant care

Just over 20% of respondents reported frequent use of existing guidelines for spine care. As noted in Table 3, there was wide variation among respondents regarding which guidelines they were most likely to follow. Barriers to CPG utilization (Table 4) included lack of CPG integration into the EHR (*n* = 7, 26%) and limited access to the guidelines (*n* = 5, 19%). A substantial majority (*n* = 21, 78%) expressed appreciation for prompts as a facilitator for CPG usage when deviating from guidelines or if they were achieving suboptimal patient outcomes. (Supplementary File 2, Question 24).

**Table 3:** Clinical practice guideline use reported by spine care clinicians

	**Guideline**	**Response**^**a**^** (%)**
Provided Responses	North American Spine Society	13 (65%)
	American College of Physicians (Low Back Pain)	10 (50%)
	Centers for Disease Control and Prevention (Opioid Prescribing)	10 (50%)
	Joint Commission (Pain Management)	9 (45%)
	Food and Drug Administration Education Blueprint (Management/Support of Patients with Pain)	7 (35%)
Open Response	Spine Intervention Society	2 (10%)
	American Association of Neurological Surgeons	2 (10%)
	Congress of Neurological Surgeons	2 (10%)
	American Academy of Orthopaedic Surgeons	1 (5%)
	National Institute for Health Care Excellence	1 (5%)
	Journal of Orthopaedic & Sports Physical Therapy	1 (5%)

^a^Responses were counted in reported total if participants answered “every visit” or “often”

**Table 4:** Barriers to clinical practice guideline use reported by spine care clinicians

Barrier to Guideline Use	Response (%)
Lack of EHR facilitation	7 (25.9%)
Limited access to clinical practice guidelines	5 (18.5%)
Misalignment with patient treatment preferences	4 (14.8%)
Other^a^	4 (14.8%)
Disagreement with recommendations	1 (3.7%)
N/A: Frequent Guideline Use	6 (22.2%)

^a^Open ended responses are summarized using qualitative analysis in text

### Spine care workforce

**Fig. 1:**
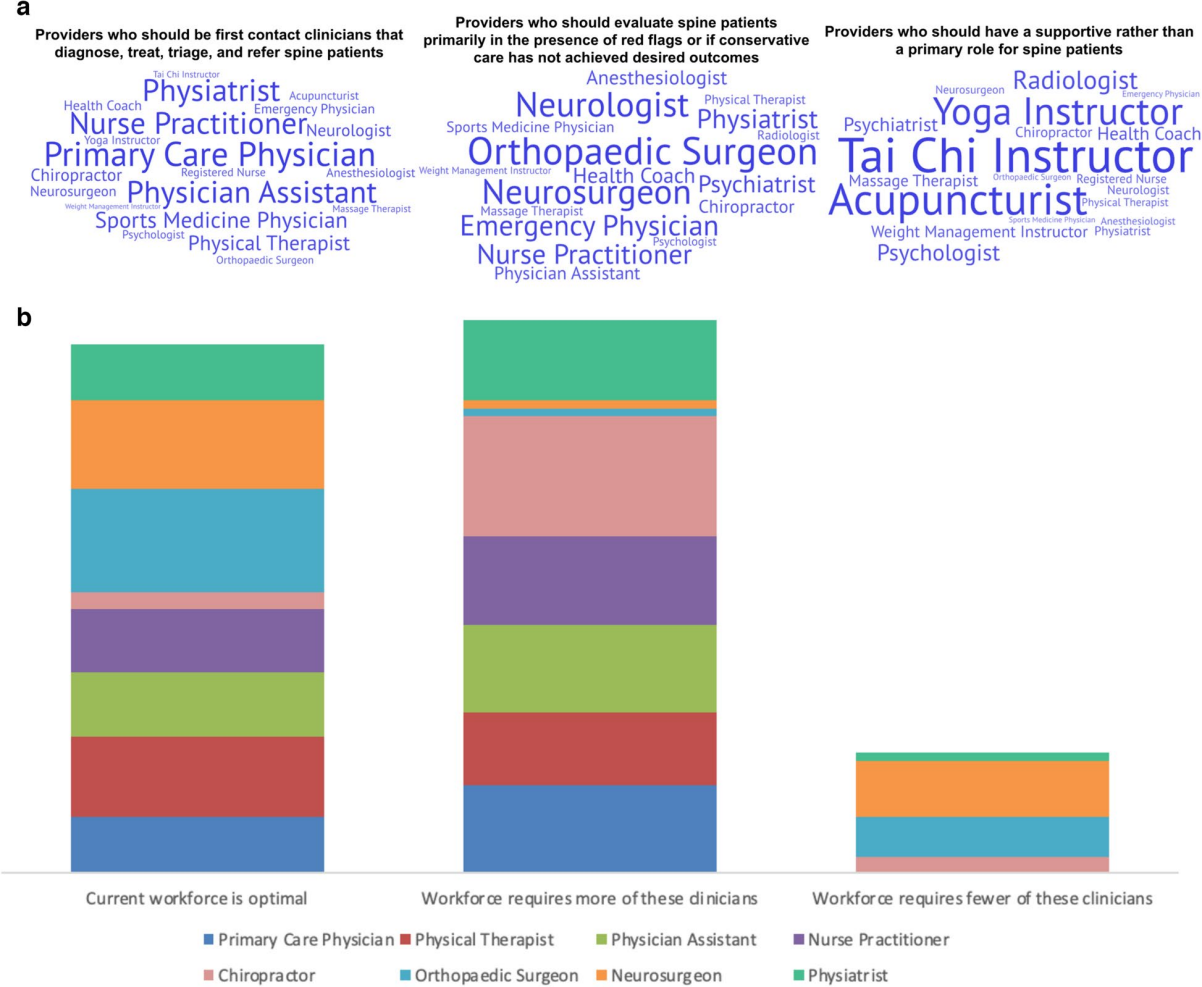
**a** Word clouds representing results from domain 6 questions (spine care workforce) regarding the roles of different providers in spine care delivery, with word size indicating the frequency of each response in the survey. Larger words correspond to responses that were mentioned more frequently, while smaller words represent less common responses. **b** Results from questions domain 6 questions (spine care workforce) regarding the optimal DUHS spine care workforce

Respondents identified the following as first-line clinicians who are best suited for the initial diagnosis, treatment, triage, and referrals for spine care patients; primary care physicians (*n* = 20), physician assistants (*n* = 16), nurse practitioners (*n* = 15), and physiatrists (*n* = 13). Neurologists (*n* = 16) and orthopaedic surgeons (*n* = 16) were viewed as the clinicians best suited to evaluate spine patients with red flags or when NPTs are ineffective. Finally, respondents considered acupuncturists and Tai Chi instructors to be best suited for a supportive role in spine care delivery. Results are demonstrated in Fig. 1a.

Figure 1b displays respondent perspectives of an optimal spine care workforce within DUHS. Clinicians where the current workforce was considered optimal included orthopedic surgeons (*n* = 13), neurosurgeons (*n* = 11), and physical therapists (*n* = 10). In contrast, the current workforce required more chiropractors (*n* = 15), followed by primary care physicians, physician assistants, and nurse practitioners (*n* = 11). Respondents tagged neurosurgeons (*n* = 7) and orthopedic surgeons (*n* = 5) as clinicians where there might be an oversupply in the workforce.

### Qualitative results

Qualitative analysis yielded 6 themes regarding comprehensive spine care: 1) Patient expectations/satisfaction, 2) Clinician experiences, 3) Comprehensive/multidisciplinary spine care, 4) Appropriate role of surgery, 5) Referral processes/patient access, and 6) Administrative concerns. Patient expectations and satisfaction emerged as influential drivers of clinicians’ treatment decisions.

*“Nowadays patients demand the care that they need and lots of physicians received patients’ complaints for not ordering tests or prescribing opioids that they want. We need more backup from higher up people in our organization and patients’ representatives to be able to drive to value-based care”.*

Clinician experiences acknowledged the challenge of applying guidelines developed for populations to the complex needs of individual patients who seek care in academic healthcare settings. The significance of comprehensive, multidisciplinary spine care was recognized alongside challenges in optimizing such approaches.

*“If I recommend an ancillary service like PT, acupuncture, chiropractic care, or injection I should be able to get the patient seen within a few days, not have to make them jump through hoops only to get a visit scheduled 6 weeks down the road…need to streamline referrals.”*

The role of spinal surgery for LBP care was facilitated by appropriate patient selection and well-established referral pathways.

*“Our triage system from emergency room (ER) and through neurosurgical (NSU)/Ortho hotlines should be more biased towards understanding what patients need and want, including a greater inclusion of physiatry and non-surgeon intake mechanisms until they are ready to consider surgery.”*

Streamlined referral processes and timely access to ancillary services were underscored as vital facilitators for effective spine care.

*“If I recommend an ancillary service like PT, acupuncture, chiropractic care, or injection I should be able to get the patient seen within a few days, not have to make them jump through hoops only to get a visit scheduled 6 weeks down the road.”*

Administrative concerns included a call for increased support from leadership and patient representatives in driving value-based care initiatives. Collectively, these themes provide valuable insights into clinician perspectives, guiding the refinement of the proposed spine care model for enhanced delivery.

## Discussion

The results from this survey of spine care clinicians in one academic healthcare system are consistent with previous work [31, 32] on the importance of addressing barriers to the implementation for optimal spine care delivery, which emphasizes multidisciplinary care approaches. Again, consistent with previous reports in the literature, we also identified a need to educate clinicians and patients on the use of guideline concordant treatment approaches [14, 33], such as the recommended use imaging, opioids, and non-pharmacological treatments such as physical therapy and spinal manipulation [12, 34–37]. Finally, spine care providers who completed our survey desired to optimize the workforce to include an interdisciplinary team of clinicians with the appropriate expertise to evaluate and treat patients with spine-related disorders. This finding is also aligned with recent conversations as to how to improve spine care delivery [27, 38].

While most clinicians at DUHS consider referrals to PSP clinicians beneficial for patients, nearly half lack confidence in effectively getting patients into these programs. The limited evidence on physician referrals to PSP clinicians complicates the ongoing problem of finding the right clinician, for the right patient, at the right time [15]. Clinicians who can intervene early and are knowledgeable about non-pharmacological spine care are needed. More than 90% of respondents reported that it would be beneficial for patients to have increased access to non-pharmacological spine care such as physical therapy or chiropractic care. Further, when respondents were asked which clinician types were lacking at DUHS, chiropractors were ranked the highest. However, neither chiropractic nor physical therapy were ranked highly in any of the three categories pertaining to the sequencing of patient interactions with clinician types. This warrants consideration given that these primary spine practitioners are well equipped to deliver first-line treatments aligned with recommendations from CPGs. Prior research indicates that patients may be less likely to transition from acute to chronic LBP if they receive NPTs at the onset of their healthcare journey [39].

There is strong support from CPGs for the utilization of PSP clinicians as a first step for LBP patients [40]. Implementation of PSPs may prevent early exposure to guideline non-compliant care, such as early imaging and opioid prescriptions, which can play a role in the development of chronic LBP [9, 10, 39]. Other approaches, like screening with the STarT Back tool to stratify patients based on prognosis, face challenges in improving referrals and further emphasize the need for different strategies [41–45]. Stepped care models that emphasize early intervention and evidence-informed, patient-centered care present potential for successful referrals to a range of clinicians recommended by CPGs [46].

An important dichotomy that requires further consideration is the disconnect between our respondents’ willingness to adapt to guideline-concordant care, and the fact that many patients do not receive this care [40, 47, 48]. There appears to be a missed opportunity for clinicians to utilize the abundant resources available at most academic healthcare systems through referrals to first-line clinicians recommended by CPGs. This discrepancy may arise due to physician’s lacking specific training in spine examination and treatment, [16] while patients may have inadequate information about high-quality spine care. Additionally, patient expectations of treatment processes, such as radiographs and medications, often diverge from these guidelines and place excessive burden on clinicians to deliver care that aligns with patients’ desires rather than care that aligns with clinical guidelines [49–51]. Existing evidence demonstrates that overcoming breakdowns in communication between physicians, NPT clinicians, and patients can be accomplished through mutual feedback to facilitate successful referrals as well as patients taking an active role in their care [52]. Educating both patients and clinicians on how to actively engage in their care and communicate effectively can facilitate the alignment of CPGs and patient expectations.

Most survey respondents were open to the idea of benchmarking; however, over half answered that they may not change their current practice even if they were aware of their lack of optimal benchmark scores. This finding is consistent with previous studies identifying gaps between guideline recommendations and actual clinical practice. Reasons for this discord range from lack of clinician knowledge regarding how to advise on recommended treatments not taught in medical school to time constraints that limit their ability to delve into the psychosocial issues often associated with spine-related disorders [16, 53, 54]. Benchmarking and other health system-level strategies to improve care quality in musculoskeletal service delivery require additional research [40, 55]. The prospect of changing practice habits is shown to be higher when decisions are supported by health systems with measures like payment adjustments, order restrictions, and the development of EHR integration for clinician guidance [56, 57]. One illustrative example is the recent adoption of a LBP imaging policy by Blue Cross and Blue Shield (BCBS) which restricts reimbursement for imaging services that are billed within 28 days of a principal diagnosis of uncomplicated LBP [58]. Though this policy change appears to be a step in the right direction, longitudinal data will be needed to determine if a demonstrable effect will be seen in the actions of clinicians and patients.

Additional findings indicated that over two-thirds of respondents expressed a willingness to change their practice regarding opioid prescribing. This agreement may be driven by the growing support from insurers and health systems for guideline-concordant opioid practices due to the ongoing public health impact of these medications across the United States [59]. Quantity limits and legislation in some states further drive the shift towards evidence-based opioid practices [60]. The major pharmacy benefit manager CVS/Caremark announced their intention to institute similar limits on initial prescriptions [60]. This system-level support suggests that health systems and insurers can play a crucial role in in influencing clinician practices to align with evolving public health concerns and guideline concordant care.

## Strengths and limitations

The strengths of this project include using an extant model of multidisciplinary spine care to develop the survey and the relative completeness of responses. The generalizability of our findings is limited, as all respondents were recruited from a small convenience sample in 1 department within a larger academic healthcare system. Healthcare workers who did not complete the survey may espouse different beliefs about the barriers and facilitators of guideline-concordant spine care and what constitutes an optimal workforce in the local healthcare setting. Coverage and sampling errors may have occurred. For example, primary care clinicians working outside the spine department, but who refer to the department, might respond differently to the survey. Additionally, we are not able to present results by clinician type due to the small sample size, our desire to offer confidentiality to respondents, and a survey platform that did not allow deeper subgroup analysis, a constraint that should be acknowledged when interpreting findings. While the survey was pretested, it was not validated, and respondents may not have understood the meaning of the questions, which may have contributed to our modest response rate. Future research should consider the use of focus groups to explore more specific facilitators to care and offer a deeper understanding of the nuances within different healthcare settings.

## Conclusion

Large healthcare systems are increasingly focused on the need to transition from fee-for-service models of spine care delivery that emphasize low value care. One approach to addressing this problem is the addition of non-pharmacological therapies, such as physical therapy and chiropractic, especially at the forefront of the patient experience. However, such movements are limited by the lack of insight regarding the perceptions of stakeholders within these settings, particularly physicians [22, 23]. Such information is required to competently navigate the intricacies involved with reshaping healthcare delivery models for spine care delivery from the current state to more comprehensive strategies. Moreover, such interdisciplinary approaches require development that goes beyond mere showcasing of the clinical value of NPTs. Rather, efforts are needed that foster a shared awareness of the benefits such interdisciplinary pathways offer to patients, clinicians, and healthcare systems. In spite of its limitations, this study takes us one step closer in this direction.

### Supplementary Information

**Additional file 1.** STROBE Statement—checklist of items that should be included in reports of observational studies.

**Additional file 2.**

## Data Availability

Data analyzed during this study are included in this published article and Supplementary file 1.

## Abbreviations

BCBS: Blue cross blue shield
CPG: Clinical practice guideline
DUHS: Duke University Health System
ER: Emergency room
HCS: Healthcare systems
IQR: Interquartile range
LBP: Low back pain
MD: Medical Doctor
NPT: Non-pharmacologic treatment
NSU: Neurosurgical
PA: Physician assistant
PSP: Primary spine practitioner
PT: Physical therapist
STROBE: Strengthening the Reporting of Observational studies in Epidemiology
