# Supplementary material for: Healthcare provider perspectives on integrating a comprehensive spine care model in an academic health system: a cross-sectional survey
Source: BMC Health Serv Res. 2024 Jan 23;24:125. doi: 10.1186/s12913-024-10578-z (PMC10804504; doi:10.1186/s12913-024-10578-z)
Supplement: Supplementary file 1 — Additional file 1. STROBE Statement—checklist of items that should be included in reports of observational studies. [file 12913_2024_10578_MOESM1_ESM.docx]

STROBE Statement—checklist of items that should be included in reports of observational studies

|  | Item No. | Recommendation | Page  No. | Relevant text from manuscript |
| --- | --- | --- | --- | --- |
| **Title and abstract** | 1 | (*a*) Indicate the study’s design with a commonly used term in the title or the abstract | 1 and 2 | Title: **Healthcare provider perspectives on integrating a comprehensive spine care model in an academic health system: a cross-sectional survey** |
|  |  | (*b*) Provide in the abstract an informative and balanced summary of what was done and what was found | 2 | Methods: 26 spine care clinicians (55% response) completed a 25-item online survey via Qualtrics on barriers and facilitators to delivering guideline concordant care for low back pain patients. Data analysis included descriptive statistics and content analysis.  Results: Respondents reported that guidelines were implementable within the HCS, but no spine care guideline was used consistently across provider types. Guideline access and integration with electronic records were barriers to use. Respondents (91.7%) agreed most patients would benefit from non-pharmacological therapies such as physical therapy or chiropractic before receiving specialty referrals. Providers perceived spine patients expected diagnostic imaging (95.6%) and medication (82.6%) over non-pharmacological therapies. Providers agreed that receiving imaging (91.6%) and opioids (69.5%) benchmarks could be helpful but might not change their ordering practice, even if nudged by best practice advisories. An optimal spine care workforce would require more chiropractors and primary care providers and fewer neurosurgeons and orthopedists. In qualitative responses, respondents emphasized the following barriers to guideline-concordant care implementation: patient expectations, provider confidence with referral pathways, timely access, and the appropriate role of spine surgery. |
| Introduction | | | |  |
| Background/rationale | 2 | Explain the scientific background and rationale for the investigation being reported | 3/4 | “Comprehensive, sustainable, patient-centered approaches to spine care are an imperative to address the global burden of back and neck pain.”  “Early intervention, which may be most efficiently provided by primary spine practitioners (PSP) such as physical therapists and doctors of chiropractic, may avert or delay the progression of spinal disability through timely diagnoses and provision of evidence-based treatments.”  “Yet, little is known about how physicians and other primary care providers view spine care pathways in these settings. Furthermore, there is a lack of information about non-pharmacologic treatments (NPT) and spine care practices in United States within private healthcare settings, and a need for a better understanding of how they are integrated into usual medical practices.” |
| Objectives | 3 | State specific objectives, including any prespecified hypotheses | 4 | “The goal of this effort was to better understand healthcare clinician perceptions of potential barriers and facilitators to the integration of guideline-concordant spine care services for patients with low back pain (LBP) at Duke University Health System (DUHS). The overall purpose was to create new knowledge while simultaneously laying the groundwork required to develop and implement the Duke Spine Health Program - an interdisciplinary, evidence-based, patient-centered model for spine care delivery, with a focus on LBP.” |
| Methods | | | |  |
| Study design | 4 | Present key elements of study design early in the paper | 4 | “We administered a cross-sectional online survey…” |
| Setting | 5 | Describe the setting, locations, and relevant dates, including periods of recruitment, exposure, follow-up, and data collection | 4-6 | “…to practicing spine care providers within DUHS between July 15 and August 10, 2021.”  “DUHS is an integrated academic health system located within the Raleigh-Durham area of North Carolina, USA. DUHS has approximately 25,000 employees and offers a full range of inpatient and outpatient clinical services, including both primary and specialty care. In the year that this study was conducted, patients paid DUHS hospitals and clinics more than 4.7 million visits, the vast majority for outpatient visits.”  “The survey was administered using Qualtrics, a secure web-based platform for data collection. Participants were contacted via email and provided a unique link to access the survey. Participants were informed that study participation was voluntary, anonymous, and all responses were confidential.” |
| Participants | 6 | (*a*) *Cohort study*—Give the eligibility criteria, and the sources and methods of selection of participants. Describe methods of follow-up  *Case-control study*—Give the eligibility criteria, and the sources and methods of case ascertainment and control selection. Give the rationale for the choice of cases and controls  *Cross-sectional study*—Give the eligibility criteria, and the sources and methods of selection of participants | 5 | “The Duke Spine Division coordinates care between the Departments of Orthopaedic and Neurosurgery. All clinicians within the Division were invited to participate in the survey, as well as four physical therapists serving in the PSP role and two primary care physicians who were actively involved in program implementation.” |
|  |  | (*b*) *Cohort study*—For matched studies, give matching criteria and number of exposed and unexposed  *Case-control study*—For matched studies, give matching criteria and the number of controls per case |  |  |
| Variables | 7 | Clearly define all outcomes, exposures, predictors, potential confounders, and effect modifiers. Give diagnostic criteria, if applicable |  | N/A |
| Data sources/ measurement | 8* | For each variable of interest, give sources of data and details of methods of assessment (measurement). Describe comparability of assessment methods if there is more than one group | *5/6* | “Instrument” paragraph |
| Bias | 9 | Describe any efforts to address potential sources of bias |  | N/A |
| Study size | 10 | Explain how the study size was arrived at |  | N/A |

Continued on next page

| Quantitative variables | 11 | Explain how quantitative variables were handled in the analyses. If applicable, describe which groupings were chosen and why | 6/7 | “Descriptive statistics summarized the data. Frequencies and percentages were reported for categorical variables, and means and standard deviations (or median and interquartile ranges (IQR)) were reported for continuous variables.” |
| --- | --- | --- | --- | --- |
| Statistical methods | 12 | (*a*) Describe all statistical methods, including those used to control for confounding |  | As above |
|  |  | (*b*) Describe any methods used to examine subgroups and interactions |  | N/A |
|  |  | (*c*) Explain how missing data were addressed |  | N/A |
|  |  | (*d*) *Cohort study*—If applicable, explain how loss to follow-up was addressed  *Case-control study*—If applicable, explain how matching of cases and controls was addressed  *Cross-sectional study*—If applicable, describe analytical methods taking account of sampling strategy |  | N/A |
|  |  | (*e*) Describe any sensitivity analyses |  | N/A |
| Results | | | | |
| Participants | 13* | (a) Report numbers of individuals at each stage of study—eg numbers potentially eligible, examined for eligibility, confirmed eligible, included in the study, completing follow-up, and analysed | 7 | “Forty-seven clinicians, including all faculty of the Duke Spine Division and key program stakeholders, were invited and 26 participated, resulting in a response rate of 55%.” |
|  |  | (b) Give reasons for non-participation at each stage |  | N/A |
|  |  | (c) Consider use of a flow diagram |  | N/A |
| Descriptive data | 14* | (a) Give characteristics of study participants (eg demographic, clinical, social) and information on exposures and potential confounders | 7/8 | Table 1 |
|  |  | (b) Indicate number of participants with missing data for each variable of interest |  | N/A |
|  |  | (c) *Cohort study*—Summarise follow-up time (eg, average and total amount) |  | N/A |
| Outcome data | 15* | *Cohort study*—Report numbers of outcome events or summary measures over time |  | *N/A* |
|  |  | *Case-control study—*Report numbers in each exposure category, or summary measures of exposure |  | *N/A* |
|  |  | *Cross-sectional study—*Report numbers of outcome events or summary measures |  | *Table 2, 3a, and 3b* |
| Main results | 16 | (*a*) Give unadjusted estimates and, if applicable, confounder-adjusted estimates and their precision (eg, 95% confidence interval). Make clear which confounders were adjusted for and why they were included |  | N/A |
|  |  | (*b*) Report category boundaries when continuous variables were categorized |  | N/A |
|  |  | (*c*) If relevant, consider translating estimates of relative risk into absolute risk for a meaningful time period |  | N/A |

Continued on next page

| Other analyses | 17 | Report other analyses done—eg analyses of subgroups and interactions, and sensitivity analyses | 6/7  11/12 | “Answers from open-ended questions were reviewed as qualitative results and coded for common themes using conventional content analysis.”  “Qualitative Results” paragraph |
| --- | --- | --- | --- | --- |
| Discussion | | | | |
| Key results | 18 | Summarise key results with reference to study objectives | 12 | “The results from this survey of spine care clinicians in one academic healthcare system underscore the importance of addressing barriers to the implementation for optimal spine care delivery. We also identified a need to educate clinicians and patients on the use of guideline concordant treatment approaches, and a desire to right size the workforce to include a more diverse mix of clinicians with the appropriate expertise to evaluate and treat patients with spine-related disorders.” |
| Limitations | 19 | Discuss limitations of the study, taking into account sources of potential bias or imprecision. Discuss both direction and magnitude of any potential bias | 15/16 | “Strengths and Limitations” paragraph |
| Interpretation | 20 | Give a cautious overall interpretation of results considering objectives, limitations, multiplicity of analyses, results from similar studies, and other relevant evidence | 13-15 | Entire pages |
| Generalisability | 21 | Discuss the generalisability (external validity) of the study results | 15/16 | “Strengths and Limitations” paragraph |
| Other information | |  | | |
| Funding | 22 | Give the source of funding and the role of the funders for the present study and, if applicable, for the original study on which the present article is based | 17 | Declarations |

*Give information separately for cases and controls in case-control studies and, if applicable, for exposed and unexposed groups in cohort and cross-sectional studies.

**Note:** An Explanation and Elaboration article discusses each checklist item and gives methodological background and published examples of transparent reporting. The STROBE checklist is best used in conjunction with this article (freely available on the Web sites of PLoS Medicine at http://www.plosmedicine.org/, Annals of Internal Medicine at http://www.annals.org/, and Epidemiology at http://www.epidem.com/). Information on the STROBE Initiative is available at www.strobe-statement.org.
