# Supplementary material for: Healthcare provider perspectives on integrating a comprehensive spine care model in an academic health system: a cross-sectional survey
Source: BMC Health Serv Res. 2024 Jan 23;24:125. doi: 10.1186/s12913-024-10578-z (PMC10804504; doi:10.1186/s12913-024-10578-z)
Supplement: Supplementary file 2 — Additional file 2. [file 12913_2024_10578_MOESM2_ESM.pdf]

	Question	Question Type	Response Options (if applicable)
	**Demographics**		
1	Age	Open response	N/A
2	Gender	Multiple choice	Male<br>Female<br>Non-binary/third gender<br>Prefer to not answer
3	Primary place of work (ZIP code)	Open response	N/A
4	Clinical Degree	Multiple response	Medical Doctor (MD)<br>Physician Assistant (PA)<br>Physical Therapist (PT)<br>Chiropractor
	If your degree is not included above, please list here	Open response	N/A
5	Clinical Department	Multiple response	Anesthesiology<br>Neurosurgery<br>Orthopaedics<br>Physical Medicine & Rehabilitation<br>Primary Care<br>Other<br>Urgent Care<br>Emergency Department<br>Neurology<br>Psychiatry<br>Integrative Medicine<br>Pain Management
	If your department is not included above, please list here	Open response	N/A
6	Clinical Setting	Multiple response	PDC Outpatient Clinic<br>Hospital Based Clinic<br>Inpatient Care

7	Occupation	Multiple response	Administrator<br>Researcher<br>Clinician<br>Professor/Educator<br>Other
8	Years in your profession	Multiple choice	0 to 3 years<br>4 to 10 years<br>More than 10 years
9	Tenure at Duke	Multiple choice	0 to 3 years<br>4 to 10 years<br>More than 10 years
10	Did you complete a fellowship in spine surgery?	Yes/No	Yes<br>No
	**Stepped Care**		
1	Patients in my clinical area would benefit from increased access to conservative approaches to spine care, such as physical therapy and chiropractic.	Likert Scale Matrix	Strongly Disagree<br>Disagree<br>Agree<br>Strongly Agree
2	Evidence-based spine care pathways are commonly followed in DUHS.		
3	Clinical care pathways are too difficult to implement and/or sustain in DUHS.		
4	DUHS has methods in place to support coordinated multidisciplinary care for spine patients.		
5	Primary care providers should recommend physical therapy before referring to specialty care.		
6	Primary care providers should recommend non-pharmacological spine care, such as yoga,		

	massage, and chiropractic, before referring to specialty care.		
7	I know how to refer patients to self-care programs, such as yoga, exercise, and weight loss, within DUHS.		
	**Resources**		
8	DUHS provides access to the full range of services needed by spine care patients in our community.	Likert Scale Matrix	Strongly Disagree<br>Disagree<br>Agree<br>Strongly Agree
9	I wish I had more resources to support me in making referrals for spine care patients in my practice.		
10	I need more information about non-pharmacological care to integrate this into my practice.		
11	I need more information about community resources for patients with spine conditions.		
12	I feel like the administrative insurance processes (i.e. benefits and authorization) are a barrier to my patient's care.		
13	Most patients expect to receive diagnostic imaging as part of their spine care treatment.		
14	Most patients expect to receive medication as part of their spine care treatment.		
15	Most patients expect to receive physical therapy as part of their spine care treatment.		
16	Most patients expect to receive chiropractic care as part of their spine care treatment.		
	**Benchmarking Performance**		

17	DUHS places too much emphasis on specialty care, such as surgery and injections, for spine patients.	Likert Scale Matrix	Strongly Disagree<br>Disagree<br>Agree<br>Strongly Agree
18	I am comfortable with my imaging ordering information being shared among providers in my division.		
19	I would reconsider some imaging requests if I knew my imaging order volume was substantially higher than my colleagues.		
20	I would reconsider some opioid prescribing if I knew my opioid prescribing volume was substantially higher than my colleagues.		
21	I am likely to give my imaging order a second thought if I see a Best Practice Advisory.		
	**Guideline Concordant Care**		
22	How frequently do you use this clinical practice guideline to guide clinical decision making:<br>• North American Spine Society: Evidence-Based Clinical Guidelines for Multidisciplinary Spine Care: Diagnosis and Treatment of Low Back Pain (Kreiner D.S., Matz P; 2020)<br>• American College of Physicians: Noninvasive Treatments for Acute, Subacute, and Chronic Low Back Pain: A Clinical Practice Guideline From the American College of Physicians (Qaseem A. et. al; 2017)<br>• CDC: Guideline for Prescribing Opioids for Chronic Pain (Dowell D. et al; 2016)	Likert Scale Matrix	Never<br>Infrequently<br>Often<br>Every Visit

	• Joint Commission: Advisory on Nonpharmacologic and Non-opioid Solutions for Pain Management (2018)<br>• FDA: Education Blueprint for Health Care Providers Involved in the Management or Support of Patients with Pain (2017)		
	Are there other guidelines you follow that are not listed here?	Open response	N/A
23	Reason(s) why I do not use clinical practice guideline for spine care frequently	Multiple response	• Electronic Health Record (Maestro Care) does not facilitate the use of clinical practice guidelines.<br>• I do not agree with the recommendations in current clinical practice guidelines.<br>• I do not have access to clinical practice guidelines.<br>• The guidelines do not align with the treatment preferences of my patients<br>• Other<br>• N/A: I use guidelines frequently
	If you selected 'other' please provide the reason(s) why you do not use clinical practice guidelines.	Open response	N/A
24	How would you feel about being nudged or educated when not following guidelines or when patient outcomes fall below guideline concordant care?	Multiple Choice	Really Appreciate<br>Somewhat Appreciate<br>Somewhat Not Appreciate<br>Really Not Appreciate
	**Spine Care Workforce**		
25	The following providers might be part of a multidisciplinary healthcare team for spine care.	Card sort grid	Acupuncturist<br>Anesthesiologist<br>Chiropractor

	Please select the group that is most appropriate for each provider:<br>• Providers who should be first contact clinicians that diagnose, treat, triage, and refer spine patients<br>• Providers who should evaluate spine patients in the present of red flags or if conservative care has not achieved desired outcomes<br>• Providers who should have a supportive rather than a primary role for spine patients		Emergency Physician<br>Health Coach<br>Massage Therapist<br>Neurologist<br>Neurosurgeon<br>Nurse Practitioner<br>Orthopaedic Surgeon<br>Physiatrist<br>Physical Therapist<br>Physician Assistant<br>Primary Care Physician<br>Psychiatrist<br>Psychologist<br>Radiologists<br>Registered Nurse<br>Sports Medicine Physician<br>Tai Chi Instructor<br>Weight Management Instructor<br>Yoga Instructor
26	Please sort each provider into 1 of the 3 boxes to indicate what is needed for an optimal DUHS spine care workforce:<br>• Current workforce is optimal<br>• Workforce requires more of these clinicians<br>• Workforce requires fewer of these clinicians	Card sort grid	Primary Care Physician<br>Physical Therapist<br>Physician Assistant<br>Nurse Practitioner<br>Orthopaedic Surgeon<br>Neurosurgeon<br>Physiatrist<br>Chiropractor
